# Supplementary material for: The genome sequence of a muscid fly, Lispocephala verna (Fabricius, 1794) (Diptera: Muscidae)
Source: Wellcome Open Res. 2026 Jun 27;11:394. [Version 1] doi: 10.12688/wellcomeopenres.26934.1 (PMC13555080; doi:10.12688/wellcomeopenres.26934.1)
Supplement: Peer review history

# Reviewer report: Reviewer response for version 1

**DOI:** 10.21956/wellcomeopenres.29653.r160420

**Published:** 2026-07-23

**Authors:** Cheng Sun

**Affiliations:** 1 Capital Normal University, Beijing, Beijing, China

*Lispocephala verna* (Fabricius, 1794) is a muscid fly classified within the diverse genus *Lispocephala*. The genome presented here represents the first available genomic data for the tribe **Coenosiini**, offering a valuable resource for phylogenomic studies within the Muscidae family. Additionally, it will facilitate comparative genomic analyses that investigate adaptations associated with predatory behavior in both adult and larval stages.

Are sufficient details of methods and materials provided to allow replication by others?

Yes

Is the rationale for creating the dataset(s) clearly described?

Yes

Are the datasets clearly presented in a useable and accessible format?

Yes

Are the protocols appropriate and is the work technically sound?

Yes

Reviewer Expertise:

insect genomics, genome evolution

I confirm that I have read this submission and believe that I have an appropriate level of expertise to confirm that it is of an acceptable scientific standard.

# Reviewer report: Reviewer response for version 1

**DOI:** 10.21956/wellcomeopenres.29653.r160419

**Published:** 2026-07-22

**Authors:** Rajib Deb

**Affiliations:** 1 ICAR-National Research Centre, Guwahat, Assam, India

Manuscript may be strengthen in inclusion of the following aspects:

 1. Include  the  functional genome annotation in terms of gene prediction, repeat annotation, and key gene families to enhance the utility of the genome assembly.

 2. Expand the discussion section on the biological and evolutionary significance of the genome with respect to  predatory behavior and comparative genomics.

 3. Briefly outline the  future area for identifying sex chromosomes, such as sequencing male specimens or comparative genomic analyses.

Are sufficient details of methods and materials provided to allow replication by others?

Yes

Is the rationale for creating the dataset(s) clearly described?

Yes

Are the datasets clearly presented in a useable and accessible format?

Yes

Are the protocols appropriate and is the work technically sound?

Yes

Reviewer Expertise:

Genomics

I confirm that I have read this submission and believe that I have an appropriate level of expertise to confirm that it is of an acceptable scientific standard.
